# Performance of Human and Porcine Derived Acellular Dermal Matrices in Prepectoral Breast Reconstruction: A Long-term Clinicaland Histologic Evaluation

**DOI:** 10.1093/asj/sjae175

**Published:** 2024-09-30

**Authors:** Allen Gabriel, G Patrick Maxwell, Erin O’Rorke, John R Harper

## Abstract

**Background:**

Human acellular dermal matrices (ADMs) remain the most used matrices in prosthetic breast reconstruction. However, the availability and cost of ADMs limit their use in prepectoral reconstruction—which requires large amounts of ADM—and alternative matrices are therefore being explored.

**Objectives:**

The aim of this study was to demonstrate the safety and efficacy of human-porcine ADM constructs via clinical outcomes and histologic evidence of graft integration.

**Methods:**

Consecutive patients undergoing tissue-expander/implant reconstructions with human-porcine ADM constructs were included. Biopsies of both ADMs were obtained at expander/implant exchange and evaluated for cellularization, vascularization, and inflammation. Postoperative complications were retrieved from patient records.

**Results:**

Fifty-nine patients met the inclusion criteria. Mean [standard deviation] follow-up was 6.7 [0.56] years; minimum follow-up was 5 years. Any complication rate was 8.6%, including skin necrosis (6.9%), seroma (1.7%), expander/implant exposure (1.7%), and return to the operating room (2.6%). A total of 138 ADM biopsy specimens were obtained from 38 patients at expander/implant exchange. Histologic analyses revealed lower fibroblast infiltration and vascularization and higher inflammatory response in porcine vs human ADM specimens, consistent with published results in nonhuman primates. Despite these differences, there were no cases of graft rejection, capsular contracture, or expander/implant loss.

**Conclusions:**

Porcine ADM performs clinically in a similar manner to human ADM, albeit with minor differences in cellular ingrowth and vascularization, suggesting that it may be an alternative to human ADM in prepectoral breast reconstruction.

**Level of Evidence: 3:**

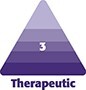

Over the last 25 years, there have been 2 paradigm shifts in prosthetic breast reconstruction: the introduction of acellular dermal matrices (ADMs) as a component of breast reconstruction^1-3^, and the shift back to subcutaneous prosthesis placement, now termed prepectoral reconstruction.^4^

ADM was introduced in 2005 as an adjunct in a widely used modification of the partial muscle coverage technique of subpectoral breast reconstruction.^1-3^ In this technique, ADM is sutured at the lower one-third of the device to provide the additional support needed at the inferior pole. Lower pole ADM placement minimizes complications associated with subcutaneous coverage without restricting lower pole expansion. Currently, almost 60% of prosthetic breast reconstructions are performed with the assistance of ADMs.^5^

The introduction of ADM for breast reconstruction has also led to the feasibility of prepectoral prosthetic reconstruction.^4^ The matrix typically covers the entire anterior surface of the device, and often the posterior surface as well, where it plays a critical role in providing a layer of vascularized regenerative tissue between the device and mastectomy flap, supporting and stabilizing the device, and allowing for precise control of the prepectoral pocket. Because of its simplicity in eliminating the need to elevate the pectoralis major muscle, attenuating the attendant problems of muscle elevation, and the preservation of the natural anatomic state of the breast with the prosthesis over the pectoralis muscle, prepectoral reconstruction is gaining in popularity, constituting almost 50% of prosthetic reconstructions.^6^

The first ADMs were of human origin.^1-3^ Since then several other ADMs of bovine and porcine origin have been developed and used in breast reconstruction.^7^ Nonetheless, human ADM remains the most used matrix.^8,9^ Because human ADMs are derived from donated cadaveric tissue, their availability can be limited by supply issues and their use limited by associated elevated costs. Given that large quantities of ADM are required for the prepectoral approach, the supply and cost limitations may be a deterrent to prepectoral breast reconstruction.^4^ Thus, alternative matrices without these limitations are being explored for prepectoral reconstruction, at the same time ensuring that the effectiveness of soft tissue coverage and patient safety are not compromised.

We have previously reported on the use of a bioabsorbable mesh, P4HB (GalaFLEX, Galatea Surgical, Inc., Lexington, MA), as a partial replacement for human ADM (AlloDerm, AbbVie Inc., North Chicago, IL), in prepectoral reconstructions.^10^ Here, we report on the use of a porcine-derived ADM, Artia (AbbVie Inc.), as a partial replacement for human ADM in prepectoral reconstructions. The purpose of this study was to demonstrate the safety and efficacy of this human-porcine ADM construct via clinical outcomes and histologic evidence of graft integration.

## METHODS

### Study

This is a retrospective study of consecutive patients undergoing immediate ADM-assisted, prepectoral, tissue-expander/implant breast reconstruction following mastectomy and who received both human and porcine ADMs in the same breast. Patients undergoing delayed reconstruction, hybrid procedures (implant and latissimus flap), revision reconstruction, or direct-to-implant reconstruction were excluded. Reconstructions were performed between May 2015 and November 2017 in the first author's practice. The study was approved by PeaceHealth Southwest Medical Center's IRB (Vancouver, WA).

### Reconstruction

**Figure 1.**
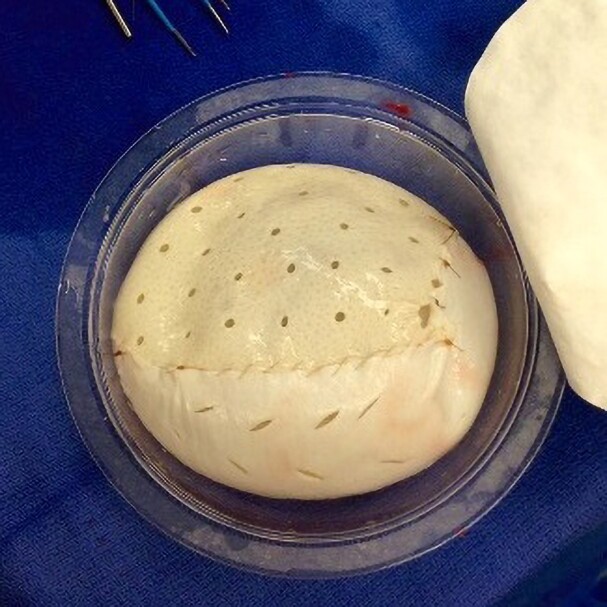
Expander partially wrapped with ADMs. Human ADM covered the upper half of the expander on the anterior surface and about 20% to 40% of the posterior surface depending on the pliability of the matrix (ie, how far it stretched). Porcine ADM covered the lower half of the expander anteriorly and laterally, including the gutter at the inferior mammary fold. ADM, acellular dermal matrix.

Prepectoral breast reconstruction was performed per the authors’ published protocol.^11^ Essentially, following mastectomy, ADMs were prepared according to the manufacturers’ recommendations. Mastectomy skin flap was accessed with indocyanine green angiography. Tissue expanders were wrapped with 2 pieces of ADM before placement in the prepectoral space. A large contour piece of perforated human ADM (10.7 cm × 21.5 cm) was placed on the upper half and a large contour piece of porcine ADM (10.7 cm × 21.5 cm) was placed on the lower half of the anterior surface of the expander (Figure 1). Porcine ADM was fenestrated prior to placement. Because human ADM is inherently elastic, it was stretched to wrap around the posterior of the expander, covering approximately 40% to 60% of the posterior surface. At the lower pole, porcine ADM covered the 3- to 5-cm gutter at the inframammary fold as well as the lateral surface of the expander to help minimize prosthesis displacement. The expanders were secured with the medial tab using 0-Vicryl sutures (Ethicon Inc., Raritan, NJ), and the matrices were secured to the inframammary fold and subcutaneous tissues with spiral 0-PDO sutures (STRATAFIX, Ethicon Inc., Raritan, NJ). Expanders (full height/variable projection) were filled intraoperatively with saline to approximately 40% of the volume, depending on flap viability, and 2 drains were placed before incision closure. Negative-pressure therapy (Prevena Incision Management System, 3M, St Paul, MN) was applied for incisional wound management for 1 week. Tissue expansion, if needed, was commenced, usually 14 to 21 days postoperatively, allowing for wound healing. Second-stage expander/implant exchange was typically performed 3 months postoperatively in nonirradiated patients, or 3 to 6 months after completion of irradiation.

### Histology

At second-stage implant exchange, on average between 3 and 4 months after first-stage reconstruction, punch biopsies of both ADMs from the anterior surface (at the breast meridian and central aspect of the pocket at the anterior flap) were obtained from 38 patients. Biopsies were only obtained from the first cohort of patients due to cost. Two biopsies were obtained from each breast. Biopsy specimens were fixed in formalin and embedded in paraffin. Samples were stained with hematoxylin-eosin (H&E) using standard methods. Further, additional specimens were subjected to immunoperoxidase staining for platelet/endothelial cell adhesion molecule-1 (PECAM-1) (formerly known as CD31).^12^ PECAM-1/CD31 is expressed constitutively on vascular endothelial cells, as well as on platelets and most leukocytes, and is commonly used as a marker to identify blood vessels in tissue sections.

### Data Collection and Analyses

Patient records were reviewed and data on demographics (ie, age and BMI), comorbidities (ie, smoking status, diabetes, and hypertension), neoadjuvant/adjuvant radiotherapy/chemotherapy use, and postoperative complications following reconstruction (ie, skin necrosis, seroma, hematoma, surgical-site infection, expander/implant exposure or loss, return to operating room, and capsular contracture) were retrieved. Major surgical-site infections and skin necroses were defined as those requiring a return to the operating room.

H&E-stained biopsy specimens were assessed for fibroblast ingrowth (cellularization), capillary ingrowth (vascularization), foreign-body reaction (presence of foreign-body giant cells), and resorptive inflammation. Capillary ingrowth was confirmed by PECAM-1/CD31 staining, if necessary. The fibroblast cell density within each graft was scored at 3 levels: high (>75%), medium (25%-75%), and low (<25%). These levels of cell ingrowth were chosen because they give a general sense of the degree of fibroblast migration into the graft without detailed quantification. Because histological sections were scored, the reviewer could easily stratify the specimens into roughly 3 levels of cellularity. Vascularization and inflammation were scored as positive or negative according to the presence or absence of capillaries or inflammatory cells, respectively. Inflammation was scored based on focal inflammatory cell density and cell morphology. The presence of foreign-body giant cells (hyperphagocytic macrophages) was used to identify a foreign-body response, which is similar to capsule formation around breast implants.

Data are presented as counts and percentages. Differences between histology findings were evaluated using the chi-square test, with statistical difference set at *P* < .05.

## RESULTS

### Study Participants

A total of 59 patients met the inclusion criteria and were included in this study. Table 1 lists the baseline demographics, comorbidities, and neoadjuvant/adjuvant therapies of these patients. At the time of surgery, the mean age of patients was 53 years (range, 26-78 years). Their mean BMI was 27.6 kg/m^2^ and 41% of them were obese, with a BMI ≥30 kg/m^2^. Almost all patients (57) had bilateral mastectomy (97%); 2 patients (3%) had unilateral mastectomy. A total of 116 breasts were reconstructed. Radiotherapy before or after first-stage expander reconstruction was uncommon (4.3% of breasts). Chemotherapy, in contrast, was prevalent with almost 36% of patients receiving this therapy, 25% before and 10% after first-stage reconstruction.

**Table 1.** Demographics, Comorbidities, and Neoadjuvant/Adjuvant Therapy Variables

Characteristic/variable	Value
Patients	59
Breasts	116
Age (years)	53 [13] (range, 26-78)
BMI (kg/m^2^)	27.6 [5.8] (range, 18-41)
Smokers	0 (0)
Diabetes (controlled)	6 (10.2)
Hypertension (controlled)	13 (22.0)
Obesity^a^	24 (40.7)
Previous breast surgery	5 (4.3)
Radiation	5 (4.3)
Preoperative (before Stage 1)	1 (0.9)
Postoperative (after Stage 1)	4 (3.4)
Chemotherapy	21 (35.6)
Preoperative (before Stage 1)	15 (25.4)
Postoperative (after Stage 1)	6 (10.2)

Values are n, mean [standard deviation] or n (%). ^a^BMI ≥30 kg/m^2^.

### Postoperative Complications and Outcomes

**Figure 2.**
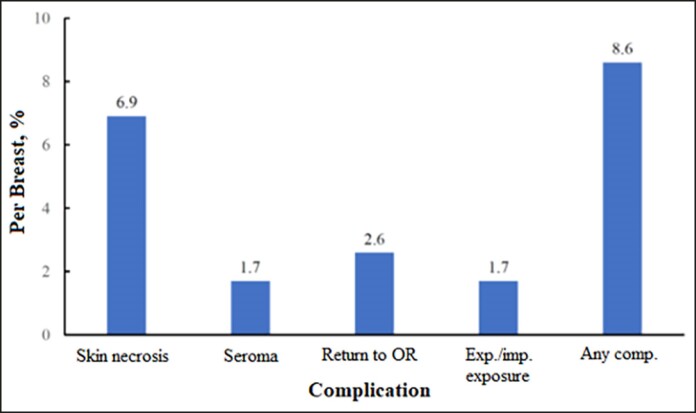
Postoperative complications.

**Figure 3.**
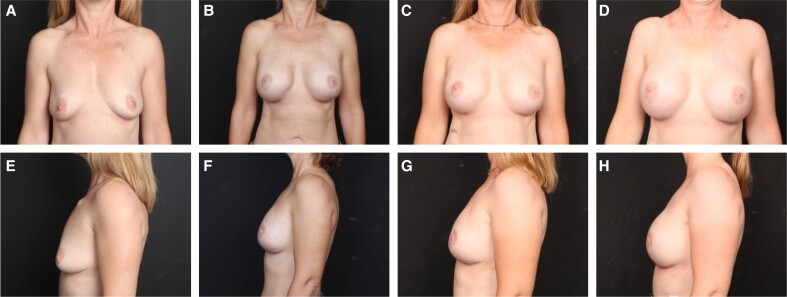
A 50-year-old female with cancer of her right breast. (A, E) Preoperative; (B, F) at 1 year, following 2-stage prepectoral reconstruction with Natrelle 495-cc 410 FX implants (Allergan, Irvine, CA); and (C, G) at 4 years postreconstruction with no additional surgeries. (D, H) The patient 4 years following implant exchange to Natrelle 560-cc SSF implants. In (G) and (H), the patient is 58 years old, 8 years postimplantation of porcine and human acellular dermal matrices.

**Figure 4.**
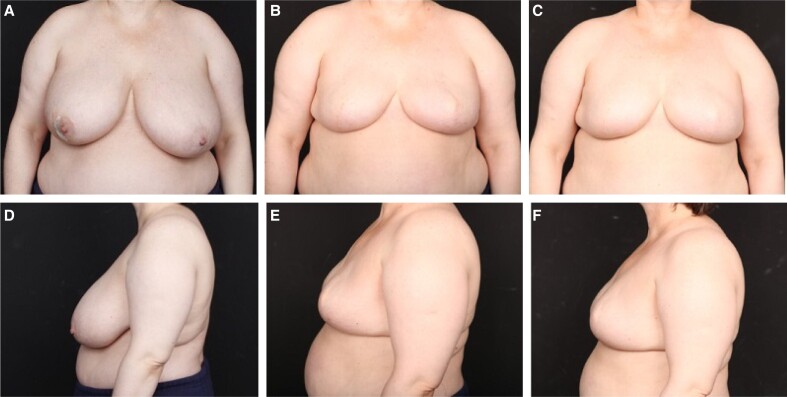
A 50-year-old female with cancer of her right breast. (A, D) Preoperative; (B, E) at 1 year following 2-stage prepectoral reconstruction with Natrelle 445-cc SCM implants; and (C, F) at 3 years postreconstruction with no additional surgeries. In (E) and (F), the patient is 53 years old, 3 years postimplantation of porcine and human acellular dermal matrices.

**Figure 5.**
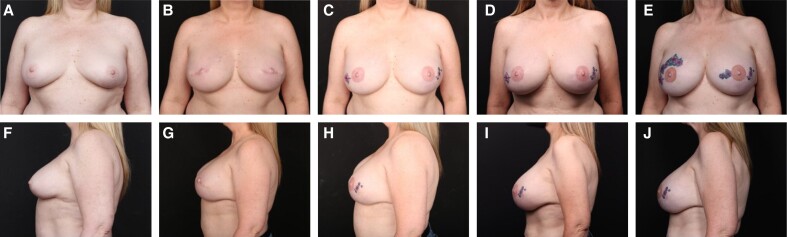
A 49-year-old female with cancer of her right breast. (A, F) Preoperative; (B, G) at 1 year following 2-stage prepectoral reconstruction with Natrelle 700-cc SRX implants; (C, H) and at 3 years postreconstruction with additional fat grafting × 3. (D, I) The patient at 6 years postreconstruction, having lost 70 pounds in weight. (E, J) The patient is 8 years postreconstruction with no additional surgeries since her third fat grafting 6 years previously (E, J). In (I) and (J), the patient is 57 years old, 8 years postimplantation of porcine and human acellular dermal matrices.

Postoperative complications occurred in 10 breasts for an any-complication rate of 8.6% (Figure 2), within a mean [standard deviation] follow-up period of 6.7 [0.56] years (range, 6.13-8.72 years). The minimum follow-up period was 5 years. Complications included 8 cases of skin necrosis (6.9%), 2 cases of seroma (1.7%), 2 cases of expander/implant exposure (1.7%), and 3 cases of return to the operating room (2.6%). Of the 8 cases of skin necrosis, 3 were major, requiring a return to the operating room. Of these 3 cases of major skin necrosis, 2 also had prosthesis exposure, and these prostheses were salvaged. The 2 cases of seroma were resolved in the office. There were no incidences of surgical-site infection, hematoma, clinically significant capsular contracture (Grade 3 or 4) in nonradiated breasts, or expander/implant loss (reconstructive failure). There were 2 cases of caspular contracture in radidated breasts (1.7%). All complications occurred within the first year of follow-up. Representative outcomes of long-term follow-ups of patients are shown in Figures 3-5.

### Histology Findings

Biopsies were obtained from 38 of the 59 patients at second-stage reconstruction: 31 had bilateral biopsies from both human and porcine ADMs, 3 had only bilateral biopsies from porcine ADM, and 4 had unilateral biopsies from human and porcine ADMs. This represents a total of 138 biopsy specimens: 66 human and 72 porcine ADMs. These were the totals used in calculating the values in Table 2. The mean time from mastectomy to second-stage implant exchange was 13.5 [7.2] weeks (range, 4-39 weeks). The earliest implant exchange/biopsy was at 4 weeks and the latest was at 39 weeks in a patient who had postmastectomy radiation in 1 breast.

**Table 2.** Histology Results: Quantification of Revascularization and Recellularization

	Porcine ADM (Artia) (n = 72)	Human ADM (AlloDerm) (n = 66)	*P*-value
Fibroblast ingrowth			
Total	59 (81.9)	63 (95.5)	.013^a^
High	20 (33.9)	28 (44.4)	
Medium	19 (32.2)	20 (31.7)	
Low	20 (33.9)	15 (23.8)	
Inflammatory cells			
Present	32 (44.4)	17 (25.8)	.022^a^
Capillaries			
Present	26 (36.1)	48 (72.7)	.00002^a^

Values are n (%). ADM, acellular dermal matrix. ^a^Statistically significant at *P* < .05.

H&E staining of biopsy specimens revealed fibroblast ingrowth in 95.5% of the human grafts and 81.9% of the porcine grafts (Table 2). Fibroblast cell density was scored as high in 44.4%, medium in 31.7%, and low in 23.8% of the human grafts. In contrast, fibroblast cell density in porcine graft was scored as high in 33.9%, medium in 32.2%, and low in 33.9% of grafts, indicating a slight shift toward a lower cell density compared with human graft. The difference in fibroblast ingrowth between the 2 grafts was statistically significant (*P* = .013) (Table 2); however, both types of graft appeared to support cellular ingrowth. H&E staining also revealed a significantly higher presence of capillary ingrowth in human vs porcine grafts (72.7% vs 36.1%; *P* = .00002) (Table 2).

When evaluating for inflammation, only signs of inflammation within the graft (intragraft inflammation) were considered. Mild inflammation in the perigraft areas was not considered because this could be an inflammatory remnant from the surgical procedure itself. Intragraft inflammation was present in almost a quarter of human grafts compared with in almost half of porcine grafts (25.8% vs 44.4%; *P* = .022) (Table 2).

**Figure 6.**
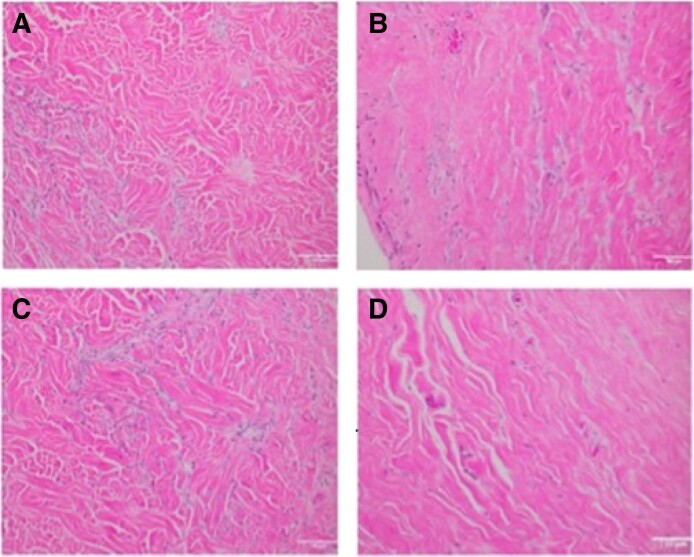
Hematoxylin-eosin–stained specimens of porcine and human ADMs. (A, C) Porcine ADM and (B, D) human ADM from (A, B) a left breast and (C, D) a right breast at 16 weeks postreconstruction from the same patient. High fibroblast ingrowth and vascularization were noted. Images were taken at 20× magnification and the scale bar represents 100 μm. ADM, acellular dermal matrix.

Representative stained biopsy specimens are shown in Figure 6 and Supplemental Figures 1 and 2.

## DISCUSSION

This study assessed the safety and efficacy of a human-porcine ADM construct as soft tissue support in patients undergoing prepectoral breast reconstruction. Safety and efficacy were evaluated clinically and histologically. Clinically, breasts reconstructed with human-porcine ADM constructs had a low rate of postoperative complications. The complication rate of 8.6% was similar to the rates we have previously reported for prepectoral reconstructions with human ADM only (7.6%) or with P4HB-human ADM constructs (6.4%).^10^ There were no cases of graft rejection, nonradiated breast capsular contracture, or expander/implant loss, suggesting that the human-porcine ADM constructs were incorporated and integrated into host tissue.

There are several ways host tissue reacts to ADM after implantation.^13^ If the ADM matrix is damaged, the host tissue will treat it as injured tissue and trigger an inflammatory response that will lead to resorption of the matrix, similar to the reaction to resorbable materials, such as PDS or Vicryl sutures. Histologically, this host reaction manifests as a hyperproliferative inflammatory response that can be identified based on cell morphology and density. If the ADM matrix is cross-linked during processing, this will make it resistant to cellular ingrowth and tissue integration. Host tissues will treat this ADM as a foreign body and wall it off, similar to capsule formation around a breast implant. In this case, the ADM matrix will remain acellular, ie, without fibroblasts, but with infiltration of multinucleated foreign-body giant cells, and perhaps the formation of a capsule around it. Finally, if the ADM matrix is recognized by the host as a native tissue scaffold, cells and vasculature will infiltrate the matrix and the matrix will be integrated into the host tissue. Ultimately, the infiltrated cells will transition into the host tissue. The tissue biopsies obtained in this study were evaluated according to these criteria. There appeared to be significant differences in the degree of fibroblast infiltration, vascularization, and inflammatory response between human and porcine ADM at the early time point of an average of 13.5 weeks after implantation. Fibroblast and capillary ingrowth were higher and inflammatory response was lower in the human grafts vs the porcine grafts. Despite these differences in biologic response between the grafts, there was no evidence of porcine graft failure, resorption, encapsulation, or rejection at the time of implant exchange/biopsy. All grafts also appeared to be integrated within the host tissue on visual inspection. Although postoperative radiotherapy can influence inflammatory response and graft integration, only 5 breasts (4.3%) were irradiated postreconstruction; hence, the influence of radiotherapy would be minimal. We postulate that the histologic differences could potentially be resolved with time, allowing porcine ADM to integrate and incorporate within the host tissue without eliciting a foreign-body response. As biopsies were not obtained at later time points, we are unable to verify this hypothesis. However, the low incidence of capsular contracture in this study over a mean follow-up of 6.7 years supports our hypothesis. Further, the biologic responses to both grafts were consistent with results we have published previously in nonhuman primates.^14-16^

The clinical findings from this study corroborate those of a recent report by Sobti et al on the use of porcine ADM for subpectoral or prepectoral reconstruction.^17^ That study reported similar complication rates between human and porcine ADM reconstructions and no significant differences in efficacy metrics, such as initial tissue-expander fill volume, ratio between initial tissue-expander fill volume and final implant size, and number of tissue-expander fills. There are, however, important differences between our study and that of Sobti et al. First, our study not only reported on clinical outcomes but also provided histologic evidence of graft integration, which is important to establish graft performance at the cellular level. Second, we used both matrices in the same breast, whereas Sobti et al used the matrices in different breasts from different patients. In using both matrices in the same breast, we have eliminated inherent biases that may impact outcome such as patient immune response; patient factors such as BMI and comorbidities; and neoadjuvant and adjuvant therapy. Third, subpectoral and prepectoral reconstructions were pooled together in the study by Sobti et al. From a technical standpoint, these 2 approaches are very different and require different amounts of ADMs. Fourth, our follow-up period was much longer. The minimum follow-up in the Sobti et al study was 6 months, whereas in our study, it was 5 years. Thus, we can conclude that the human-porcine ADM construct has minimal long-term safety concerns. Besides the study by Sobti et al, there are 2 other reports on the use of porcine ADM (Artia) in breast reconstruction in the published literature.^18,19^ In both studies, major complications were low and in one study there were no incidences of clinically significant capsular contracture over a mean follow-up of 3 years.^19^

**Figure 7.**
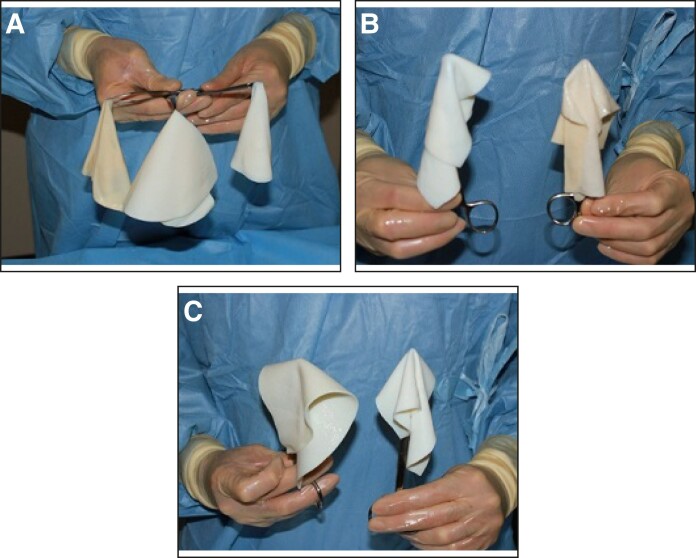
Pliability of porcine and human ADMs. (A) Human ADM (left), porcine ADM not used in this study (center), and porcine ADM used in this study (right). (B) Direct comparison of human ADM (right) and porcine ADM used in this study (left). Note the very similar drapability of both products leading to ease of use clinically. (C) Direct comparison of porcine ADMs not used in this study (right) and used in this study (left). Note the difference in drapability and stiffness of the products. ADM, acellular dermal matrix.

The porcine ADM, Artia, that we used has several attributes that are appealing for breast reconstruction. Artia is less pliable than any human ADM but more pliable than other xenografts; for example, another porcine ADM, Strattice (AbbVie Inc.)^20^ (Figure 7). Taking this difference in pliability between the matrices into consideration, we used porcine ADM at the lower pole, to minimize lower pole stretch over time, and human ADM at the upper pole. The less pliable porcine ADM at the lower pole provides for a more controlled expansion that stabilizes key breast landmarks, and may be desirable over a more liberal stretch arising from using human ADM that can lead to lower pole stretch and ptosis. The more pliable human ADM at the upper pole helps to cover a larger surface area of the expander anteriorly and posteriorly at the upper pole, thus stabilizing the expander within the pocket. Despite the difference in pliability, the drapability of the 2 matrices does not differ. Artia can be handled intraoperatively in a similar fashion to human ADM with ease of matrix placement and coverage. This porcine ADM also has a more uniform thickness from piece to piece compared with any human ADM. Consistency in thickness may help minimize variability in outcomes.

Based on the clinical and histologic evidence of a lack of a foreign-body response, we conclude that this porcine ADM (Artia) performs in a similar manner to human ADM (AlloDerm) and that it can be safely used in place of human ADM as a long-term support for implants in prepectoral breast reconstruction. It is possible that in the future this porcine ADM may be a suitable alternative to human ADM because of its inherent biomechanical and biological attributes. Lack of use of porcine ADM in the past was due to poor drapability and difficulty in handling the product. In this study, however, Artia, behaved similarly to a human ADM.

This study is limited by the retrospective design and its inherent shortcomings and the lack of histologic data from all patients and from later time points. Nonetheless, the presence of an internal control (having both matrices in the same breast), the long follow-up period (minimum of 5 years), and histologic evidence of matrix integration (absence of foreign body response) lend credence to our findings.

## CONCLUSIONS

Porcine ADM (Artia) can be safely used in 2-stage prepectoral reconstruction for prosthesis support and coverage. This is the first study that has provided both clinical and histologic evidence suggesting that this porcine ADM performs in a similar manner to human ADM (AlloDerm), which has been in use for the past 20 years. Based on its physical attributes of pliability and drapability, porcine ADM may represent a more predictable matrix to human ADM. More clinical experience is needed to better understand the benefits and limitations of porcine ADM for use in breast reconstruction.

## Supplemental Material

This article contains supplemental material located online at www.aestheticsurgeryjournal.com.

## Supplementary Material

sjae175_Supplementary_Data
