# Supplementary figures and images for: Performance of Human and Porcine Derived Acellular Dermal Matrices in Prepectoral Breast Reconstruction: A Long-term Clinicaland Histologic Evaluation
Source: Aesthet Surg J. 2024 Sep 30;45(1):56–62. doi: 10.1093/asj/sjae175 (PMC11634380; doi:10.1093/asj/sjae175)

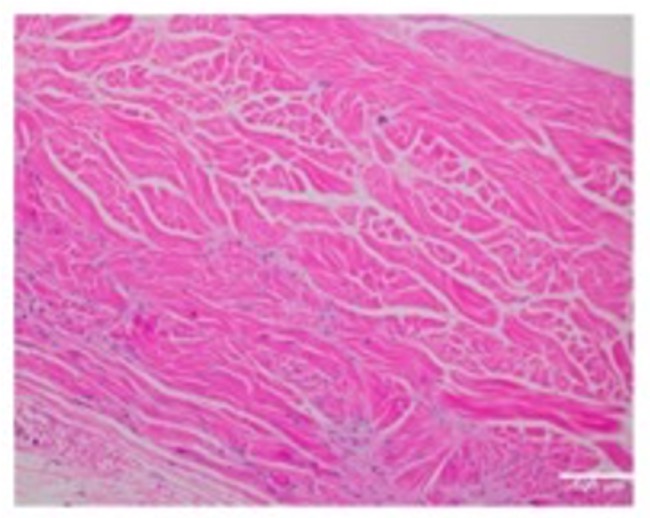

Supplement: sjae175_Supplementary_Data [file sjae175_supplementary_data.zip › SDC1A copy.jpg]

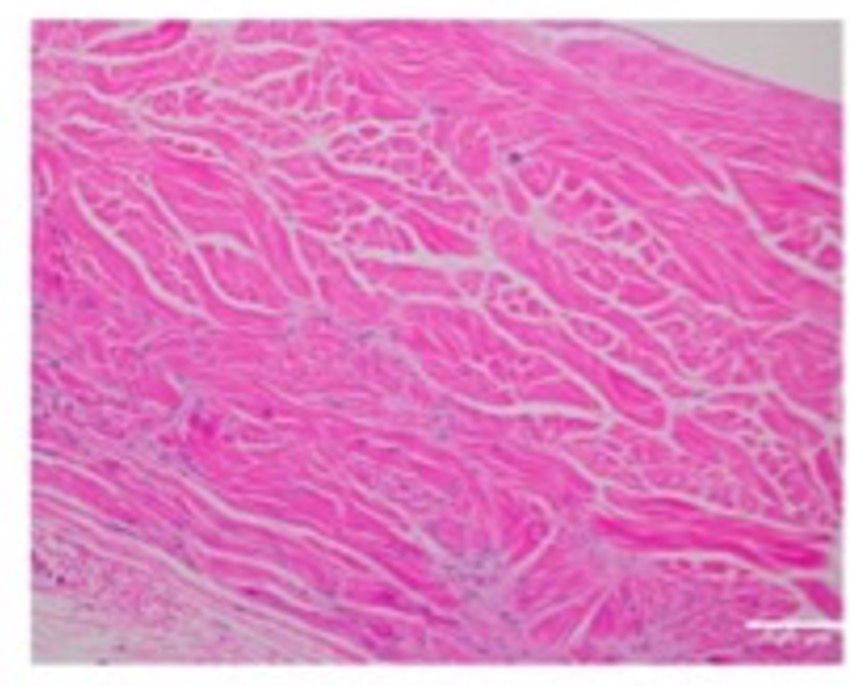

Supplement: sjae175_Supplementary_Data [file sjae175_supplementary_data.zip › SDC1B copy.jpg]

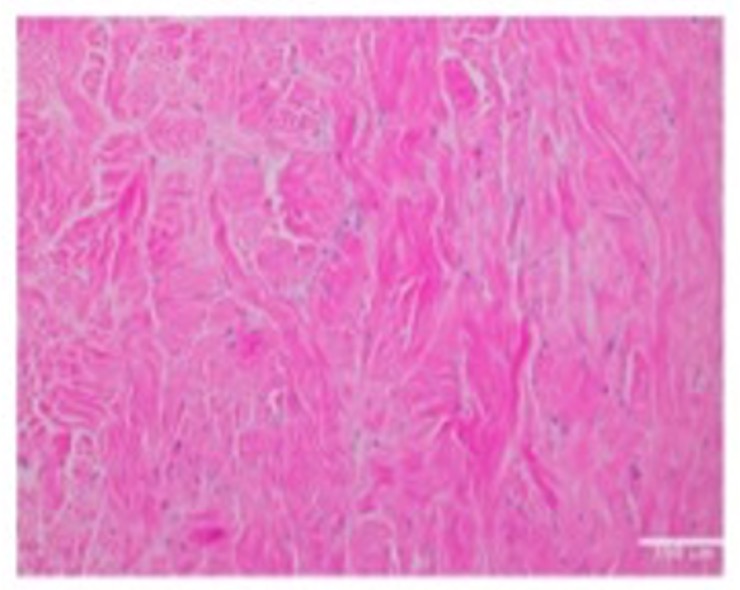

Supplement: sjae175_Supplementary_Data [file sjae175_supplementary_data.zip › SDC1C copy.jpg]

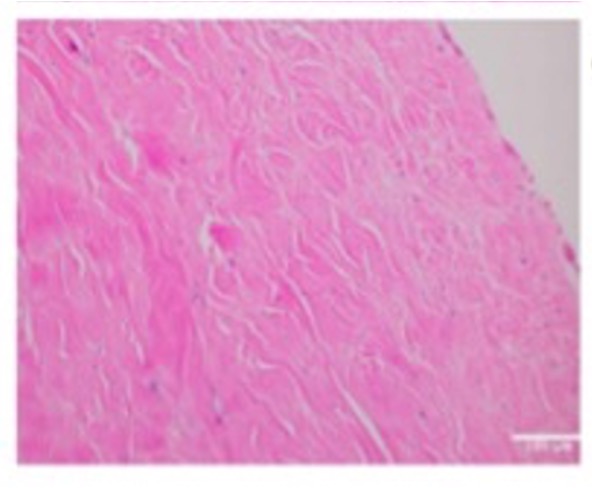

Supplement: sjae175_Supplementary_Data [file sjae175_supplementary_data.zip › SDC1D copy.jpg]

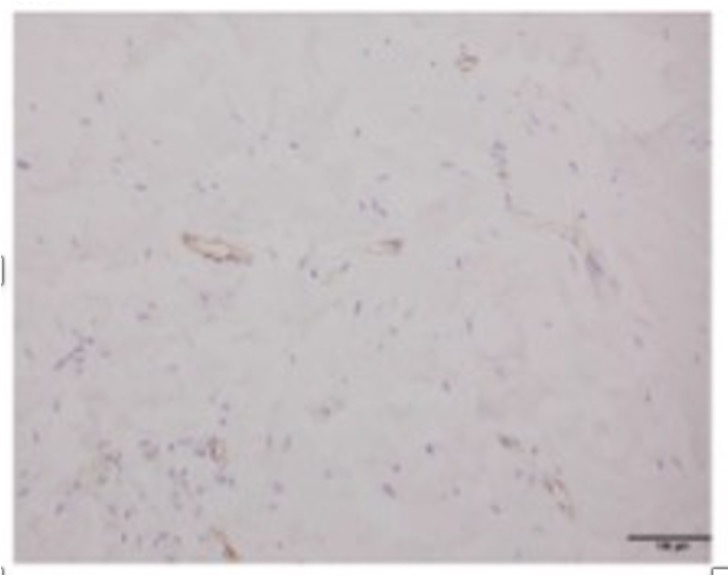

Supplement: sjae175_Supplementary_Data [file sjae175_supplementary_data.zip › SDC2A copy.jpg]

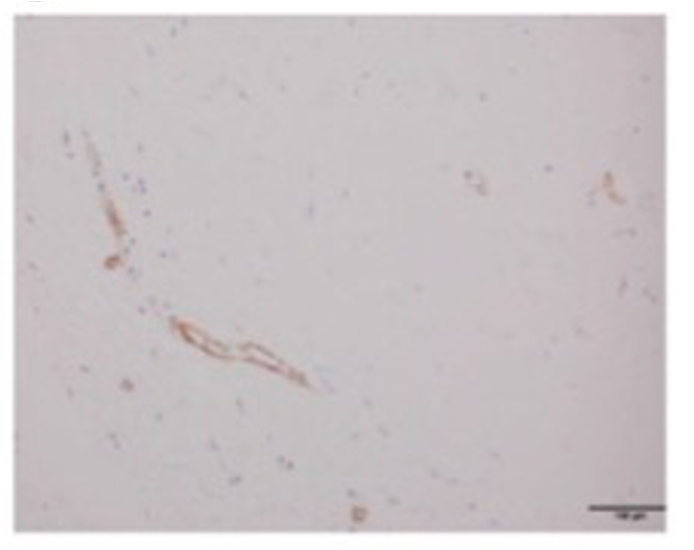

Supplement: sjae175_Supplementary_Data [file sjae175_supplementary_data.zip › SDC2B copy.jpg]
